# Development and Assessment of Professional Competences among Polish Nursing Students during a 3-Year Education Cycle Trying

**DOI:** 10.3390/ijerph19127192

**Published:** 2022-06-11

**Authors:** Magdalena Brodowicz-Król, Monika Kaczoruk, Paulina Kaczor-Szkodny, Danuta Zarzycka

**Affiliations:** 1 Department of Paediatric and Paediatric Nursing, Faculty of Health Sciences, Medical University of Lublin, 20-930 Lublin, Poland; brmagdalena@gmail.com (M.B.-K.); danuta.zarzycka@umlub.pl (D.Z.); 2 Department of Epidemiology and Biostatistics, Institute of Rural Health in Lublin, 20-950 Lublin, Poland; kaczor.paulina@imw.lublin.pl

**Keywords:** competences, nursing students, education, practice development

## Abstract

The essence of the profile of nursing professional competences are basic behavioral characteristics, as well as mastering practical skills. The aim of this study was determination of the development of professional competences of perceiving a patient by first-, second-and third-year students of licentiate nursing study. The study was of a longitudinal character, and lasted for 3 years. The research instrument used was the Ascent to Competence Scale, and traditional assessment of students’ knowledge and skills. A statistically significant relationship was observed between the average grade in practical education from all three years, and competences in student–teacher relationship. Higher grades in theoretical education were accompanied by lower results obtained by students in the area of nursing competences. The respondents’ opinions concerning the development of knowledge and professional skills were relatively high among first-year students, which may be related with a sharp increase in nursing knowledge, whereas third-year students considered it important to perceive a considerable development of professional competences. The results of this study can help in the design of education programs meeting quality standards, and alignment with students and population health needs, which is adequate to the assumptions of the WHO Global Strategic Directions for Nursing and Midwifery 2021–2025.

## 1. Introduction

### 1.1. Professional Competences

According to the dictionary definition of medical terms, competence means ‘a legal capability for performing an action and understanding its meaning’, which is substantively supplemented by Storey et al. by skills and possibilities to provide safe and effective practice [1]. Professional competences are the place of a constant and formal use of communication, knowledge, technical skills, clinical reasoning, emotions, values, and reflections in everyday practice for the good of an individual and for society. Competences are built up on the foundation of basic clinical skills, scientific knowledge, and moral development [2]. However, the World Health Organization (WHO) defines nursing competences as requirements concerning knowledge, applied attitudes, and noticeable manual and intellectual skills, which jointly create capability for delivering specialist professional care [3]. While specifying professional competences of nurses, the International Council of Nurses (ICN) additionally attracts attention to the level of actions based on the ‘effective application of a combination of knowledge, skill and judgment’ [4]. The American Nursing Association (ANA), while referring to professional competences of nurses, emphasizes that this is activity in accordance with the intention to provide ‘deliberate performance’ in care, based on integration of knowledge, skills, and personal capabilities, as well as traits obtained in both formal and informal educational experiences, including those based on reflective learning. Nursing knowledge covers all important information, including those within the scope of standards, practices, and professional effectiveness. The skills include ‘habits of mind’, and psychomotor and interpersonal skills, as well as diagnostic and ethical reasoning skills. *Habits of mind* reflect an individual pattern of thinking, problem solving, and decision making. Personal skills and traits represent these attributes, which may exert an effect on the capability of a given person for effective functioning, for example, capability for listening, honesty, a sense of knowing one’s own strengths and weaknesses, having emotional intelligence, and openness to opinions. A nurse’s capability for acting on an expected level may be associated with the character of the situation, which includes the consideration of the patient and his/her environment [5].

### 1.2. Formal Essentials of Nursing Competences

The provision of high-quality nursing care undoubtedly depends on the possessed vocational preparation [6]. In view of the constantly growing importance of the role of nursing and nurses, it was considered indispensable to improve the quality of education by creating possibilities to obtain higher vocational education approached as basic for the profession, where a graduate could obtain the vocational title of a licentiate in nursing [7]. The International Council of Nurses emphasized the importance of a constant development of nursing competences as professional responsibility and public law in order to provide high quality nursing care for all, and reasonable health policy worldwide, which is described in detail in the The WHO Global Strategic Directions for Nursing and Midwifery2021–2025 [8,9]. The general requirements concerning competences for European countries were specified in the EU Directive of 2013 on the recognition of professional qualifications. According to Directive 2013/55/EU of the European Parliament and of the Council, formal qualifications of a nurse responsible for general care should be an evidence that a given professional is capable of applying at least the following competences: independently diagnose, effectively cooperate with other entities in the health sector, strengthen individuals, families and groups in the direction of health promoting life style, independently initiate life-saving emergency measures and conduct actions in crisis and disaster situations, independently give advice and provide quality and assessment of nursing care, comprehensively communicate professionally, and cooperate with representatives of other professions in the health care sector, or competences for analysis of the quality of care in order to improve one’s own professional practice [10].

For many years, the European countries have undertaken actions to work out joint professional competences. In 2006, 21 European countries, including Poland, began efforts within the Tuning Program aimed at the development of a set of professional nursing competences. A document was issued which considered five groups of professional competences of a nurse [11]:

1. Concerning professional values and role of nurses;
2. Concerning nursing practice and making clinical decisions;
3. Cognitive;
4. Interpersonal, including communication technology;
5. Managerial, organizational—related with team work.

Despite the fact that EU Directives contain common guidelines concerning nursing education for the Commonwealth countries, educational programs for individual countries slightly differ [12].

In Poland, the document currently in effect concerning education standard of medical staff, including nurses, is the Announcement by the Minister of Education and Science of 6 April 2021 in the matter of announcement of consolidated text of the regulation by the Minister of Science and Higher Education on education standards in preparation for work as a physician, dentist, pharmacist, nurse, midwife, laboratory diagnostician, and paramedic (Journal of Laws, 2021, Item 755) [13]. According to the Act of 15 July 2011 on the occupation of nurses and midwives, nursing is an independent medical profession. The Act regulates the number of classes at school providing higher vocational studies (first-degree study). A nurse obtains professional qualifications after graduating from a nursing school on the level of first and second cycle studies. In addition, a nurse has a right to professional training within post-graduate studies in a domain applicable in health care [14].

### 1.3. Determinants of the Development of Professional Competences by Nursing Students

The process of education during first degree study at the specialty of nursing should be characterized by an educational offer of the highest quality, because the subject of education—in the area of health sciences—is of special social concern. A complex process of education is conditioned by a wide spectrum of factors, thus, the development of professional competences of students of nursing also remains under relatively varied influences. To our knowledge it has been divided in two ways:

1. According to genesis: internal, inherent in school; and external, inherent in the environment;
2. According to the direction of action of a given factor: positive, which exert an effect on the improvement of quality; and negative, which exert an effect on a decrease in this improvement.

These factors are shaped by various phenomena and processes from the social, economic, or political domains. In addition, these factors condition each other; therefore, it seems justifiable to analyze them not only from the aspect of the effect on the development of competences, but also mutual interactions [15]. Regarding internal factors, conditioning the development of professional competences should be classified in terms of those inherent in an individual pursuing the process of education, including student–teacher relationship, student’s conduct in daily life, student support during classes, the need for student’s belonging to the group, students’ openness, and the character of curriculum content [16]. The factor determining the development of professional competences may also be the area of knowledge in which the shaped competences are located [17]. The systems of motivation of students and the staff, as well as mobility of the participants of the education process, are also important. Within the factors which determine the development of professional competences is also self-awareness of the subject of the education process in terms of pro-quality orientation [18]. External factors conditioning the development of professional competences should also include the environment of the individual pursuing the process of education. In this group of factors, characteristics that may be important are: location of the place of the education process (variety of facilities providing health care), budget, type of education pursued by an individual, nursing skills of the staff, skills in the area of education, professional ethics, nursing care services, and professional development. As can be seen from the above description, a part of the factors may occur in both the first and the second group, which emphasizes their mutual connections and interactions [19].

For the purpose of the project, it was considered that the development of competences depends, to a great extent, on individual, interpersonal, contextual, and organizational elements, inseparably connected with the clinical practice of the students [20]. Analysis of literature shows that positive work and educational environments are characterized by progressive leadership, cooperation between teams, transparency in the division of tasks, loading with work in accordance with the possessed skills, mechanisms of appreciation of students, and encouragement to participate in decision making [21]. Levett-Jones and Lathlean propose conceptual framework based on studies of experiences from student internships. They suggest that students’ competences are developed by behaviors of the clinical team, which may help them in a systematic satisfaction of their social and emotional needs indispensable for learning. The researchers indicate that there are similarities between these educational needs of students and the Maslow’s hierarchy of needs, which means that the students must feel comfortable and safe, and they need the sense of acceptance, i.e., the sense of belonging, which is of a fundamental importance for their self-esteem. Such a situation provides students the possibility to apply their knowledge in practice at an individual pace, thus reaching the level of personal achievements. Summing up, Levett-Jones and Lathlean suggest that students need that their presence is known to the medical staff, and the work ordered is transparent and recognizable by everyone. Achieving this effectively can help students feel included into the therapeutic team. As members of the team, students have better chances for self-esteem. It would be best if teams were informed and managed in the area of supporting these behaviors which are crucial for learning, in order that students could achieve competences. Therefore, in the system of education which is the environment of carrying out the project of the study, during practical training, the students were introduced into the natural clinical environment by an academic teacher, whereas professional training, which constituted a half of the curriculum of training, was carried out in clinical conditions under the supervision of a nurse mentor in a health care facility [20].

### 1.4. Education Programas a Didactic Modulator in Shaping Professional Competences of Nurses

Thinking about a graduate of nursing through the prism of professional competences is a new quality of thinking about characteristics of nurses on a modern labor market. The authors of the study assumed that the research project would allow development of the attitude towards optimization of the process of shaping professional competences of nurses. Considering the importance of professional competences in education of nurses and quality of the provided care it seems right, or even expected that the attempt to assess their development should be of a longitudinal character.

An important aspect prior to detailed analyses associated with nursing competences is the presentation of the education curriculum pursued in the study group. The curriculum consisted of three parts (educational stimuli I-III), each of them lasted for 10 months, i.e., 30 didactic weeks (Table 1). After cessation of the stimulus professional competences of students were measured by student’s self-assessment, and grades from examinations and credits, together with the grade from the Virtual School collected. These grades are the consequence of evaluation of students’ knowledge and skills by academic teachers and practice mentors. In the process of evaluation, the method of a single-choice test was used, and observation of the student during performance of the task ordered.

## 2. Materials and Methods

### 2.1. Nature of the Study

In the project monitoring, studies were selected by choice, which are a type of evaluation research. Evaluation research is the type of study used for the determination of the effect of social intervention program for students for the achievement of a specified change, i.e., professional competences and collection of data which would improve the solution [21]. In the project, which is of a non-experimental character, monitoring evaluation was applied in order to trace the progress and improvement of the program. According to Richards, it may be defined as a monitoring study, i.e., indicating how a given process works in a given period of time. In addition, two main types of research are distinguished, i.e., compliance monitoring, diagnostic monitoring, and performance monitoring. The last two are of the greatest importance in this project. Such a study is usually conducted for a longer period of time, in this case for 3 years [22,23,24]. In some cases, a monitoring study requires gradual interventions, i.e., educational stimulus I, educational stimulus II, and educational stimulus III, which means that the results may change slightly when the methods of monitoring change, and changes are introduced in the investigated area [25]. This type of study was designed to overcome the existing gap between the axes of theory and practice. In this type of study, it is assumed that the researcher abandons the passive, isolated attitude, thus fulfilling the postulate of seeking a greater academic contribution into the practical world [26,27]. In the light of the above-mentioned criteria of monitoring study, the researchers’ assumption was the possibility of designing educational programs, which would satisfy quality standards, based on the results of the study obtained.

Prior to the proper study, a pilot study was carried out, which was conducted in a group of 15 students, 5 students from each year, after the winter semester. This stage of the study was completed in 2013. The students were informed about the aim of research and willingly shared their observations. Considering the result of the pilot study the form of coding the questionnaires was decided, and the scope of socio-demographic data modified.

The basic study was of a longitudinal character, and lasted for 3 years, during the period from 2014–2016, i.e., from the first until the third year of licentiate nursing study, and was conducted three times in the same group, starting from the first year and ending in the third year. All stages of the study were conducted after theoretical and practical classes, after each year at the university completed

### 2.2. Materials

To acquire nursing competency, nurses must possess: personal traits necessary for nursing, professional attitudes and behaviors, and the ability to provide care based on professional knowledge and skills [28]. Therefore, professional competences were analyzed, as well as factors which affect these competences. The undertaken research problem focused on finding a relationship between the period of studying nursing, and the development of professional competences of a nurse. The study included students of nursing (first degree) at the Medical University in Eastern-Central Europe. Final analysis included 90 respondents.

### 2.3. Objective

The aim of the study was assessment of the development of nursing competences during licentiate study, throughout the three-year education cycle, in the same group of respondents, and after each year of education grades were analyzed from examinations and credits obtained in the Virtual School system. Professional competences were analyzed, as well as factors which affect these competences. As a criterium of the development of competences analysis of school grades accompanying the process of studying was also used, which means that in the analysis of the development of competences, not only self-assessment by students was applied, but also the perspective of academic teachers contained within the evaluation of these competences.

Individual grades obtained by of the examined students were analyzed contained in the examination protocols and credits in subjects (*anatomy, physiology, genetics, microbiology, parasitology, pathology, radiology, pedagogics, law, psychology with interpersonal communication, sociology, public health, dietetics, philosophy and ethics of the nursing profession, nursing essentials, biochemistry, biophysics, English language, physical examination, primary health care, health promotion, pediatrics and pediatric nursing, pharmacology, surgery and surgical nursing, emergency medical services, internal diseases and internal medicine nursing, geriatrics and geriatric nursing, neurology and neurological nursing, midwifery, gynecology and obstetric—gynecological nursing, psychiatry and psychiatric nursing, anesthesiology and anesthesiological nursing, palliative care*) in the academic syllabus and deposited in the Virtual University system during the period 2014–2016.

### 2.4. Instruments

The research instrument applied was the Ascent to Competence Scale—ACS. Prior to the study, the ACS was adapted and validated [29]. According to MA. McCoy et al. the Ascent to Nursing Competence Scale measures the process of acquiring competences and progress on the way to achieve professional competences by nursing students [30]. The value of the ASC is the possibility to assess the knowledge and skills of a student/nurse, together with the elements of the clinical environment, which are important factors of the development of professional competences. Apart from the teacher, this is the nursing/therapeutic team which shapes students’ competences, playing a supportive role, and providing the feeling of safety. For the students, it is extremely important to be accepted by the interdisciplinary team, which is translated into the feeling of belonging to the team. Levett-Jones and Lathlean (2009) confirmed that the feeling of belonging favors the building by the students of self-confidence in professional actions [20].

The ACS scale consists of 5-point Likert scale questions, ordered in 3 subscales:

1. ACS-ST (S1–S6)—*student–teacher relationship,* consists of 6 items [Cronbach’s alpha coefficient = 0.71];
2. ACS-BW (B1–B17)—*the need for a student/nurse to belong to the group*, consists of 17 items [Cronbach’s alpha coefficient = 0.90];
3. ACS-LC (L1–L13)—*knowledge and skills of the student/nurse*, consists of 13 items [Cronbach’s alpha coefficient = 0.92].

The three areas of the adopted tool—the Ascent to Nursing Competence Scale are characterized by high criterion validity and reliability. Cronbach’s alpha coefficient for the subscale ACS—(S1–S6)—student/nurse cooperation was 0.71, subscale ACS—(B1–B17)—the need for a student/nurse to belong to the group was 0.90, while for the last subscale (L1–L13)—knowledge and skills of the student / nurse Cronbach’s alpha coefficient was 0.92.

### 2.5. Statistical Analysis

The results obtained were statistically analyzed. Normality of the distribution of variables in the examined groups was investigated using the Shapiro–Wilk test for normality. Using the analysis of variance, the results were compared in the category of conditioning and development of nursing competences, with consideration of the following subscales: ACS-ST, ACS-BW, and ACS-LC in the subsequent years of study. The results of documentation in the subsequent years were compared using the Friedman’s ANOVA.

To check the relationship between variables, Spearman rank correlation r was used. Spearman rank correlation coefficients were used to investigate the relationship between the development of nursing competences, and school grades obtained by students and mean grade in the three-year education cycle.

*t*-Student test was used to compare differences between two groups of variables. Using *t*-Student test, the results in theoretical and practical education were compared, student–teacher relationship (ACS ST), student’s belonging to the group, and (ACS BW) between respondents with lower and higher nursing competences, based on data collected after completion of each year of education.

In order to select respondents who differed by nursing competences the k-means clustering was performed.

In the analysis, the level of significance was set at *p*<0.05 indicating the existence of statistically significant differences or relationships. The database was created and statistical analyzes were performed based on the software Statistica 9.1 (StatSoft, Cracow, Poland).

### 2.6. Bioethical Aspects

The participation of the students in the study was voluntary, while maintaining anonymity. The students completed the questionnaire independently, without the presence of the teachers. The duration of completing the questionnaire was approximately 15 min. The respondents’ replies were adjusted based on initials of the name and surname, and the date of birth. In addition, if the data were not complete, the respondents’ handwriting in the questionnaire was compared.

The documents concerning the participation in the study were kept maintaining full discretion for the protection of respondents’ personal data, only the researchers connected with the project had access to the data. In the study, the principles contained in the Declaration of Helsinki were observed. In order to obtain an access to the electronic system of students’ grades—Virtual University, consent was obtained from the Dean of the Faculty of Health Sciences, Medical University, Lublin.

The study was carried out after obtaining consent from the Bioethical Committee of the Medical University of Lublin No. KE-0254/112/2014.

## 3. Results

The majority of respondents participating in the study were females. During the first year at university, 103 students participated in the study, consisting of95.15% females and 4.85% males. During the subsequent year, 118 respondents participated in the study, consisting of94.07% females and 5.93% males, whereas during the third year 104 students were examined, consisting of96.15% females and 3.85% males. The final study group included 90 students, from whom complete data were collected concerning three years at university, consisting of86 females (95.56% of the total number of respondents) and 4 males (4.44% of the total number of respondents); the students in the study were aged between 19–37 (age in the first year at university). The specificity of the study specialty did not allow an equal participation of respondents according to gender. The study group was homogenous with respect to age. Analysis according to age reported by the respondents in the first year at university showed that the majority were aged 20 (71.11% of the total number), while every tenth respondent was aged 21 (11.11%). The study group included also several students aged 22 (6.66%), 19 (5.56%), and few respondents aged 23 and over (5.56%). (Table 2).

Table 3 demonstrates the results of scales of the development of nursing competences of only those respondents who participated in the three-year cycle of the study. The results in the category of conditioning and development of nursing competences (ACS) possible to obtain were within the range 0–4. In none of the dimensions analyzed, statistically significant changes were observed concerning the results obtained during the subsequent years at university. With respect to student–teacher subscale (ACS-ST), the first-year students obtained the result M = 2.44, in the subsequent year—M = 2.33, and in the third year, M = 2.26. In this category a slight downward tendency was noted; however, the differences were statistically insignificant (*p* = 0.195). Considering the subscale ‘the need for a student/nurse to belong to the group’(ACS-BW), first-year students obtained the result M = 2.31, in the second year, M = 2.22, and in the third year, M = 2.26 (here, the differences were also statistically significant, *p* = 0.603). Slightly higher results were observed with respect to professional knowledge and skills (ACS-LC)—first-year students obtained the result M = 2.44, second-year students, M = 2.40, and third-year students, M = 2.51. However, these results did not significantly change statistically during the three-year education cycle (*p* = 0.552).

Table 4 presents relationships between indicators of conditioning and development of students’ competences in three diameters (student–teacher relationship, ACS-ST; student’s belonging to the group, ACS-BW; and professional knowledge and skills of the nursing student, ACS-LC) grades obtained in each year of education and during the whole three-year education cycle (also with division into theoretical and practical education). None of the analyzed relationships occurred statistically significant—the results obtained by students presenting their competences after the first, second, and third year of education are not correlated with the average grade obtained after the first, second, and third year of education. No statistically significant relationships were observed with respect to student–teacher relationship (ACS-ST), student’s belonging to the group (ACS-BW), or professional knowledge and skills (ACS-LC) of the students after each year of the study. However, a statistically significant relationship was found between average grade from practical training from all three years of the study and competences in the area of student–teacher relationship (r = −0.245, *p* = 0.044), while on the threshold of significance with student’s belonging to the group (r = −0.239, *p* = 0.050). In both cases, higher grades from theoretical education were accompanied by lower results obtained by students in the area of nursing competences; however, correlations coefficients indicate very weak relationships between the analyzed variables. The relationship between average grade from the years of theoretical education and results in professional knowledge and skills occurred to be very weak, on the threshold of significance—(ACS-LC) (r = −0.230, *p* = 0.059). Furthermore, in this case, higher grades were accompanied by lower results obtained by students in analyzed nursing competences.

By means of cluster analysis, the examined students were divided into groups according to the results in nursing competences obtained in the first, second, and third year of the study. The distinguished groups of students with various levels of competences, were compared according to the grades obtained in theoretical and practical education in the first, second, and third year of the study, and the results in student–teacher relationship component. Cluster analysis allowed the distinguishing of two groups in each year of education (Table 5).

Groups of students with various levels of competences were compared according to the grades obtained by them in theoretical and practical education in all years of the study. The study group included 90 students from whom a complete set of data from three years of research was collected. In turn, a group of 68 students for whom a complete set of relevant data was available were investigated in order to obtain the results in student–teacher relationship and student’s belonging to the group.

In the first year of the study, the first cluster included 33 students characterized by lower results concerning the analyzed variables—this group was defined as students with ‘low competences’. In turn, the second cluster included 57 respondents who obtained higher results obtained in individual scales examining nursing competences—this group was defined as students with ‘high’ competences.

In the second year, the first cluster included 40 students with lower results in the analyzed variables. The second cluster covered 50 students characterized by higher results according to the scales investigating the above-mentioned competences.

Based on the results obtained by students in the third year, 51 persons with lower results in nursing competences were selected, and 39 who obtained considerably higher results regarding variables characterizing nursing competences.

The conducted analysis did not show statistically significant differences in analyzed variables between students of the first and second years of the study assigned to the selected clusters. The respondents were characterized by similar results in education, as well as student–teacher relationship and student’s belonging to the group. The highest differences were found among third-year students, where statistically significant differences were observed between cluster of professional competences and high student’s self-esteem in the area of student–teacher relationship (ACS-ST; *t* = −3.000 *p* = 0.004), and student’s belonging to the group (ACS-BW; *t* = −2.818 *p* = 0.006).

## 4. Discussion

A study of professional competences of nurses is very important in the formation of professional identity. In order to develop a professional identity, it is necessary to improve awareness in the area of competences contributing to this professional profile, not only with respect to the main persons concerned, but also with regard to the group of recipients, which is society. According to M. Rodríguez-Pérez et al., competences ascribed by society to nursing specialists do not overlap with the set of competences described in the professional profiles of nurses, which limits the proper development of the profession [31]. The presented results of the development of professional competences of Polish students can be compared with the results obtained in the study conducted in Belgium. The results of a study S. Liou and C. Ching-Yu showed a lack of differences with respect to professional behaviors and overall competences throughout the education process; however, significant differences were found with respect to the competences which are fundamental for care (e.g., administration of drugs by oral route), and specialist nursing competences. The results of this study demonstrated that in the system of education the utmost importance is attached to the shaping of specialist psychomotor competences [32].

A statistically significant relationship confirmed between the mean grade in practice and the elements of the clinical environment, which are important factors of the development of professional competences, indicates the importance of these elements in the shaping of nursing competences evaluated by a school grade [33]. In our study, higher grades in theoretical education were accompanied by lower grades obtained by students in nursing competences. This result may be explained by insufficient use of theoretical knowledge in the practice of care, illustrated by the results of the descriptive study which clearly showed that EBP has not yet been sufficiently integrated with the nursing programs in the Polish system of education [34].

A study conducted by researchers from Lublin, Poland, and the United Kingdom concerning the concept of medical and nursing care demonstrated that students of nursing considered that their concern is both practical and emotional, and that was a major feature of their identity as nurses. In turn, students of medicine perceived the practical dimension of care as an additional activity, which may also compromise ones’ perceptions of competence together with the degree of autonomy in nursing care or expectations regarding the professional role [35]. All students emphasized the importance of possessing time for care, which focused on the attitudes of students of medicine and nursing towards ‘care’ [36]. The results of our study demonstrate a different perspective of the nursing students in the analyzed dimension of professional competences. In our study, a relationship was confirmed between the stage at the university and the development of knowledge and skills.

Interesting study results were presented by Murphy from Wales [37]. The researcher investigated the effect of nursing education on caring behaviors of nursing students during 3-year licentiate nursing study. The key finding was statistically significant difference in caring behavior of students between the first and the third year of study, the results concerning the third year were lower. Preliminary analysis showed a decrease in average grade from 3.57 down to 3.46 between students of the first and the third year at university. A decrease in standard deviation is also interesting (0.398–0.297)between students of the first and the third year. The highest result was obtained by the youngest students, aged 17–25. The process of shaping nursing competences among students is affected by an older age of nurses who carry out practical education(mean age 53.64) [38], which, as indicated by a study by Toto and Limone, exerts a negative effect on the use of modern, effective didactic methods based on advanced technologies [39]. Analysis of our results shows similar tendencies. Professional competences in the first year at the university were higher, compared to the third year. In our study, the lowest result during 3-year period was noted in the category of student’s belonging to the group. Similar results were obtained from the research of graduating nursing students in 10 European countries, which showed that older students, those with working experience in health care, satisfied with their current degree program, with excellent or good study achievements, graduating to first study choice and having a nursing career plan for future assessed their competences higher.

Referring to the study by Salamine et al., professional competences in Poland are on a higher level than in Finland. Analysis of our results indicates that among students examined after the first and the third year, the highest results in the area of development of professional competences were obtained according to the scale professional knowledge and skills [40]. The study described the concept of care perceived by students of the first year of nursing study before and after the first clinical classes. The students started their nursing education with a caring vision, both on the theoretical and practical levels. The results revealed that the students started their nursing education with a deeply humanistic vision of caring for both the conceptual and behavioral levels, as well as behavioral level of competences of subjective treatment of an individual [41].

A study conducted by Maluwa et al., dealt with the issue of professional competences also from the moral aspect. The key attributes of moral competences were, among others, kindness, compassion, caring, critical thinking, ethical decision-making ability, problem solving, honesty, and respect for people. Based on the study, it was indicated that moral professional competences may and should be used as a tool for the improvement of care in nursing practice [42]. This is also confirmed by British research, which indicates that among the requirements for nurses employed in public service, the behavioral characteristics were indicated, such as: care, compassion, determination, motivation, and commitment [43]. This aspect was also important in the self-assessment performed by the nurses who work in acute care hospital settings and relationship with personal burnout, work-related burnout, or burnout in relations with patients [44,45]. In addition, studies revealed an interesting phenomenon indicating that higher grades in theoretical education were accompanied by lower grades obtained by students in nursing competences, as well as the fact that higher grades were accompanied by lower results obtained by the respondents in the area of professional knowledge and skills. According to AM. McNelis et al., the above-presented results may be due to the fact that students often focus on pursuing tasks and adjustment to the team, at the cost of deepening, expanding, and self-regulation of learning [46]. Moreover, meta-analysis performed by M. Stoffels et al. showed that learning is a social process, which is highly dependent on the environment [47]. In order to enhance an active engagement of students in learning, a flexible, social form of self-regulation of learning is necessary, as well as the understanding of one’s own role in the system of health care [48].

The review of available literature databases showed that no similar studies have been conducted worldwide. More studies are needed to validate current new instruments and further describe the factors involved in the development of nursing competences. The Nurse Professional Competence Scale can identify professional competence areas for further development, which is important for the quality of health care. As indicated by Halabi et. al., there are significant relationships between professional competences and the quality of nursing care or patient safety [49].

In the project carried out in natural conditions of organization of the process of education, an analysis was assumed of the development of professional competences of students at the specialty of nursing from two separate perspectives, i.e., of a student in the form of self-assessment using the Ascent to Competence Scale, ACS, and traditional grades formulated by teachers. The results of the study are relatively surprising, because there are no statistically significant differences in self-assessment of the development of nursing competences by the students. Slight mathematical differences indicate the perception of a favorable change in the student–teacher relationship, which is probably related with the approach to students different from the paternalistic relationship in the secondary school system (ACS ST—student–teacher relationship). In addition, the students’ opinions regarding the development of professional knowledge and skills (mean arithmetic values for ACS-LC) were relatively high in the first year, which may be associated with a sharp growth in nursing knowledge, whereas in the third year, it was considered important to perceive a considerable development of professional competences. In future, it might be worth to more precisely analyze the methods of evaluation of the outcomes of learning applied by teachers, because the predominance of evaluation of knowledge over the evaluation of skills may constitute one of the causes of the lack of correlation between evaluations by teachers and self-assessment by students. The definition of the final outcome of education may also be questionable, because this is most probably an acquisition of partial knowledge and skills resulting from subjects, modules, and not complex competences, which would allow the performance of the profession in practice.

## 5. Strengths and Limitations of the Study

For more than a decade, researchers and practitioners dealing with education have investigated in which way innovative measures may be integrated with traditional education, in order to enrich the experience of learning and increase its effectiveness. Due to these innovative methods, it is possible to create completely new learning environments to better support teaching and learning in nursing. They emphasize flexibility of learning, effectiveness, efficiency, commitment, and reflectivity, where both theoretical and practical, formal, and informal learning are integrated. It is considered that the experience of learning may be enriched, and the effectiveness of learning increased by the adjustment of individual traits and preferences in learning, an increased degree of engagement, and feedback information, as well as guidelines concerning time and provision of contents rich in the media. Together with the occurrence of mobile and smart devices the environments of learning will function in huge heterogenous network infrastructures providing extended experiences, which may mobilize educational actions, encourage for interactive cooperation via social media, and simultaneously facilitate the collection of data associated with learning for analytical purposes. Therefore, the ACS is very useful, which, apart from the possibility of evaluation of student’s professional knowledge and skills, allows the monitoring of the student/teacher-nurse relationship, and cooperation with the therapeutic team as elements of the didactic environment.

Due to the presented monitoring research, the value and didactic effectiveness of the implemented program has been recognized, which would allow its modification in the way to optimize the use of the possessed resources in the context of anticipated effects—professional competences of students.

Three-year systematic collection of information concerning the effects of the application of the program of education in the form of evaluation of school grades by academic teachers and mentors of clinical practice, self-assessment of students in the area of knowledge and skills, and indication of opinions concerning the clinical environment exerting an effect on the development of professional competences allowed modification of the education programs. In addition, an initiative was undertaken to equip mentors in practical education with soft competences, including those within the range of interpersonal communication considering their individual traits and preferences, and modern didactic methods. This simultaneously showed the need for better preparation of higher education for reacting to crisis situations, such as pandemics, migrations, conflicts, etc. Higher education may be of a greater value in promoting the ‘importance of good well-being and quality of life’.

The study was conducted at one university, and covered a relatively small group of students of nursing, with a considerable domination of females, which may have resulted in a moderate bias of the results obtained. Extension of the study by academic teachers and mentors in practical education could contribute to the recognition of the conditioning from another perspective of shaping professional competences of students of nursing.

## 6. Conclusions

In the project carried out in natural conditions of organization of the process of education, an analysis was assumed of the development of professional competences of students at the specialty of nursing from two separate perspectives, i.e., of a student in the form of self-assessment using the Ascent to Competence Scale, ACS, and traditional grades formulated by teachers. The results of the study are relatively surprising, because there are no statistically significant differences in self-assessment of the development of nursing competences by the students. Slight mathematical differences indicate the perception of a favorable change in the student–teacher relationship, which is probably related with the approach to students different from the paternalistic relationship in the secondary school system (ACS ST—student–teacher relationship). A statistically significant relationship was observed between the average grade in practical education from all three years, and competences with respect to student–teacher relations. Higher grades in theoretical education were accompanied by lower grades obtained by the students in nursing competences. The results of students presenting self-assessment of their competences in the first, second, and third year of study are not correlated with the average school grade obtained after the first, second, and third year of education. Students with ‘low’ nursing competences were characterized by significantly lower result concerning the student–teacher relations and students belonging to the group than respondents with ‘high’ competences. In addition, the students’ opinions regarding the development of professional knowledge and skills (mean arithmetic values for ACS-LC) were relatively high in the first year, which may be associated with a sharp growth in nursing knowledge, whereas, in the third year, it was considered important to perceive a considerable development of professional competences. In future, it might be worth a more precise analysis of the methods of evaluation of the outcomes of learning applied by teachers, because the predominance of evaluation of knowledge over the evaluation of skills may constitute one of the causes of the lack of correlation between evaluations by teachers and self-assessment by students. The definition of the final outcome of education may also be questionable, because this is most probably an acquisition of partial knowledge and skills resulting from subjects, modules, and not complex competences, which would allow the performance of the profession in practice.

## 7. Implications for the Practice of Education of Nurses

Due to the above-mentioned practical profile, the system of education of nurses is, to a great extent, based on the clinical conditions of the provision of health care, which should also be the object of interest of the organizers of education. Optimization of the process of shaping professional nursing competences should include cooperation between the entities carrying out education and health care facilities. Considering the above-presented analyses the evaluation of professional competences by the traditional method is insufficient, and the use of standardized research tools is recommended assessing the level of development of these competences, e.g., the Ascent to Competence Scale, especially considering the connection between knowledge and skills, identification and belonging to the group, and interpersonal skills (relations with others). The results of this study can help in the design of education programs meeting quality standards, and alignment with population health needs, which is adequate to the assumptions of the WHO Global Strategic Directions for Nursing and Midwifery 2021–2025 [50].

## Figures and Tables

**Table 1:** Characteristics of the selected features of the curriculum in Poland.

	Educational Stimulus I	Educational Stimulus II	Educational Stimulus III
**Number of didactic hours**	1745 h	1545 h	1580 h
**Form of conducted classes**	Lectures, classes, practical training, professional internships	Lectures, classes, practical training, professional internships	Lectures, classes, practical training, professional internships
**Modules**	**Basic sciences:** anatomy, physiology, genetics, microbiology, parasitology, biochemistry, biophysics, pathology	**Basic sciences:** pharmacology	None
	**Social sciences:** pedagogy, law, psychology with interpersonal communication, sociology, public health including organization of work, English language (part I)	**Social sciences:** English language (part II)	**Social sciences:** English language (part III) (foreign languages, facultatively: German, Spanish, Russian, Italian) with prevalence of the form of classes (60 h).
	**Primary nursing care:** essentials of nursing, philosophy and ethics of nursing profession, dietetics.	**Primary nursing care:** physical examinations, primary health care (part I)	**Primary nursing care:** studies in nursing/elements of statistics and information technologies, primary health care (part II)
	**Specialist nursing care:** primary health care, health promotion	**Specialist nursing care:** internal diseases and internal medicine nursing (part I), surgery and surgical nursing (part I), pediatrics and pediatric nursing (part I)	**Specialist nursing care:** geriatrics and geriatric nursing, neurology and neurological nursing, midwifery, gynecology and obstetric-gynecological nursing, internal diseases and internal medicine nursing (part II), psychiatry and psychiatric nursing, anesthesiology and anesthesiology nursing, surgery and surgical nursing (part II), palliative care, pediatrics and pediatric nursing (part II).
**Methods of evaluation of development of professional competences**	tests of knowledge, essays, oral examination, *Objective Structured Clinical Examination* (OSCE), observation of performance of the task ordered	tests of knowledge, essays, oral examination, observation of performance of the task ordered, written examination (open questions)	Tests of knowledge, essay, oral examination, written examination (open questions), observation of performance of the task ordered

**Table 2:** Demographic characteristics of the examined group of students who participated in the 3-year study cycle.

		N	%
Gender	Females	86	95.56
	Males	4	4.44
Age *	19	5	5.56
	20	64	71.11
	21	10	11.11
	22	6	6.66
	23 and over	5	5.56
Total		90	100.00

* The mean age of students beginning the study was 21.74 years.

**Table 3:** Characteristics of conditioning and development of nursing competences (ACS-ST, ACS-BW, ACS-LC) over the 3-year education cycle on the level of first-degree study.

Variable	Year at University	Mean	SD	Statistical Analysis
**ACS-ST**	1	2.44	0.69	F (2.178) = 1.651 *p* = 0.195
	2	2.33	0.68	
	3	2.26	0.68	
**ACS-BW**	1	2.31	0.53	F (2.178) = 0.508 *p* = 0.603
	2	2.22	0.57	
	3	2.26	0.62	
**ACS-LC**	1	2.44	0.75	F (2.178) = 0.596 *p* = 0.552
	2	2.40	0.65	
	3	2.51	0.68	

Legend: F—result of one-way repeated measures ANOVA, *p*—statistical significance. ACS-ST—student–teacher relationship; ACS-BW—student’s belonging to the group; ACS-LC—professional knowledge and skills of the nursing student.

**Table 4:** Spearman’s rank correlation coefficient between the development of nursing competences and school grades obtained by students after the first, second, and third year of the study, and average grade during three-year cycle of education.

Correlated Variables		ACS-ST	ACS-BW	ACS-LC
Year I	r	−0.091	0.044	0.030
	*p*	0.459	0.719	0.809
Year I KT	r	−0.108	−0.026	0.094
	*p*	0.380	0.837	0.444
Year I KP	r	−0.062	0.081	−0.019
	*p*	0.617	0.510	0.881
Year II	r	−0.165	−0.013	−0.076
	*p*	0.179	0.916	0.538
Year II. KT	r	−0.138	−0.131	−0.070
	*p*	0.262	0.287	0.569
Year II. KP	r	−0.127	0.070	−0.054
	*p*	0.301	0.571	0.661
Year III	r	0.002	−0.116	−0.150
	*p*	0.989	0.347	0.224
Year III. KT	r	0.152	0.074	0.001
	*p*	0.215	0.551	0.991
Year III. KP	r	−0.080	−0.179	−0.180
	*p*	0.516	0.145	0.141
Average grade I–III Theoretical and practical education	r	−0.222	−0.214	−0.199
	*p*	0.069	0.080	0.104
Average grade I–III Theoretical education	r	−0.158	−0.161	−0.230
	*p*	0.199	0.189	0.059
Average grade I–III Practical training	r	−0.245	−0.239	−0.158
	*p*	0.044	0.050	0.199

Legend: ACS-ST—student–teacher relationship, ACS-BW—student’s belonging to the group, ACS-LC—professional knowledge and skills of the nursing student, Year I, II, III—average grade in the first, second, and third year of the study, Year I, II, III KT—average grade in theoretical education in the first, second, and third year of the study, Year I, II, IIIKP—average grade in practical training in the first, second, and third year of the study.

**Table 5:** Comparison of results of theoretical and practical education, student–teacher relationship (ACS-ST), student’s belonging to the group (ACS-BW) between respondents with lower and higher nursing competences, based on data collected after the first, second, third year at university.

Year I					
Variable	ACS-LC	N	M	SD	Comparison of Mean Values
**Education in general—average grade in the first year at university**	‘low’	24	4.05	0.18	*t* = 1.195 *p* = 0.236
	‘high’	44	3.99	0.22	
**Theoretical education—average grade in the first year at university**	‘low’	24	3.93	0.30	*t* = −0.116 *p* = 0.908
	‘high’	44	3.94	0.34	
**Practical education—average grade in the first year at university**	‘low’	24	4.11	0.20	*t* = 1.841 *p* = 0.070
	‘high’	44	4.01	0.21	
**ACS-ST**	‘low’	33	2.33	0.54	*t* = −1.126 *p* = 0.263
	‘high’	57	2.50	0.75	
**ACS-BW**	‘low’	33	2.27	0.56	*t* = −0.574 *p* = 0.567
	‘high’	57	2.33	0.52	
**Year II**					
**Education in general—average grade in the second year at university**	‘low’	29	4.48	0.17	*t* = −0.389 *p* = 0.698
	‘high’	39	4.50	0.18	
**Theoretical education—average grade in the second year at university**	‘low’	29	4.21	0.29	*t* = −1.059 *p* = 0.294
	‘high’	39	4.29	0.26	
**Practical education—average grade in the second year at university**	‘low’	29	4.61	0.19	*t* = 0.184 *p* = 0.855
	‘high’	39	4.60	0.19	
**ACS-ST**	‘low’	40	2.23	0.69	*t* = −1.302 *p* = 0.196
	‘high’	50	2.42	0.67	
**ACS-BW**	‘low’	40	2.19	0.55	*t* = −0.473 *p* = 0.637
	‘high’	50	2.25	0.60	
**YEAR III**					
**Education in general—average grade in the third year at university**	‘low’	40	4.74	0.17	*t* = −0.643 *p* = 0.523
	‘high’	28	4.76	0.08	
**Theoretical education—three-year average grade**	‘low’	40	4.45	0.30	*t* = 0.219 *p* = 0.827
	‘high’	28	4.44	0.26	
**Practical education—average grade in the third year at university**	‘low’	40	4.82	0.18	*t* = −0.893 *p* = 0.375
	‘high’	28	4.85	0.08	
**ACS-ST**	‘low’	51	2.08	0.68	*t* = −3.000 *p* = 0.004
	‘high’	39	2.49	0.61	
**ACS-BW**	‘low’	51	2.11	0.63	*t* = −2.818 *p* = 0.006
	‘high’	39	2.46	0.54	

Legend: N—number of respondents, M—mean, SD—standard deviation, ACS ST—student–teacher relationship, ACS BW—student’s belonging to the group, *t*—result of *t*-Student test, *p*—statistical significance.

## Data Availability

The data presented in this study are available on request from the corresponding author.
